# Epidemiology, genetics, neuroimaging, clinical features, and treatment between overall and performance-only social anxiety disorder: a narrative review

**DOI:** 10.1080/19585969.2026.2661986

**Published:** 2026-04-28

**Authors:** Lisa Okamura, Yuki Ueji, Tamae Izumi, Daisuke Fujikane, Tatsuhide Ukisu, Hiroki Murakami, Toshiki Shioiri, Kazutaka Ohi

**Affiliations:** ^a^ School of Medicine, Gifu University, Gifu, Japan; ^b^ Department of Psychiatry, Gifu University Graduate School of Medicine, Gifu, Japan

**Keywords:** Performance-only, social anxiety disorder, epidemiology, clinical features

## Abstract

Social anxiety disorder (SAD) is a common psychiatric condition marked by a significant fear of social evaluation. To better identify a meaningful subgroup, the DSM-5 introduced a “performance-only” specifier, replacing the generalised specifier in the DSM-IV. We conducted a narrative review comparing overall SAD (including generalised and performance-only forms) and performance-only SAD across epidemiology, genetics, neuroimaging, clinical characteristics, and treatment. Overall SAD affects 5–13% of the population, whereas performance-only SAD has been reported in about 0.3–0.7%. Overall SAD typically emerges in early adolescence and is more prevalent among females, whereas performance-only SAD tends to begin in mid-adolescence and is relatively more prevalent among males. Family studies indicate stronger familial aggregation in overall SAD, suggesting greater genetic contribution compared with performance-only SAD, although direct heritability estimates for the latter remain limited. Neuroimaging studies of overall SAD reveal alterations in fronto-limbic and default mode networks, whereas neurobiological data on performance-only SAD are limited and suggest more localised, context-dependent neural responses. Clinically, performance-only SAD is linked to fewer psychiatric comorbidities, less functional impairment, and higher socioeconomic status. Treatments for overall SAD typically include CBT and pharmacotherapy, such as SSRIs or SNRIs. In contrast, performance-only SAD is commonly managed with exposure-based CBT and, in some cases, beta-blockers for situational symptom relief. These findings suggest that performance-only SAD may represent a distinct subtype within the SAD spectrum, although empirical research on this specifier remains limited.

## Introduction

Social anxiety disorder (SAD) is a psychiatric disorder characterised by an intense fear of specific social situations in which individuals may be observed or judged by others. This fear often leads to avoidance behaviours or the enduring of such social situations with intense distress. In accordance with the Diagnostic and Statistical Manual of Mental Disorders, Fifth Edition (DSM-5) criteria (APA 2013), common symptoms include marked fear of negative evaluation, as well as physical symptoms such as trembling, sweating, blushing, or a racing heart. These symptoms must persist for at least six months to satisfy the diagnostic threshold.

**Figure 1.**
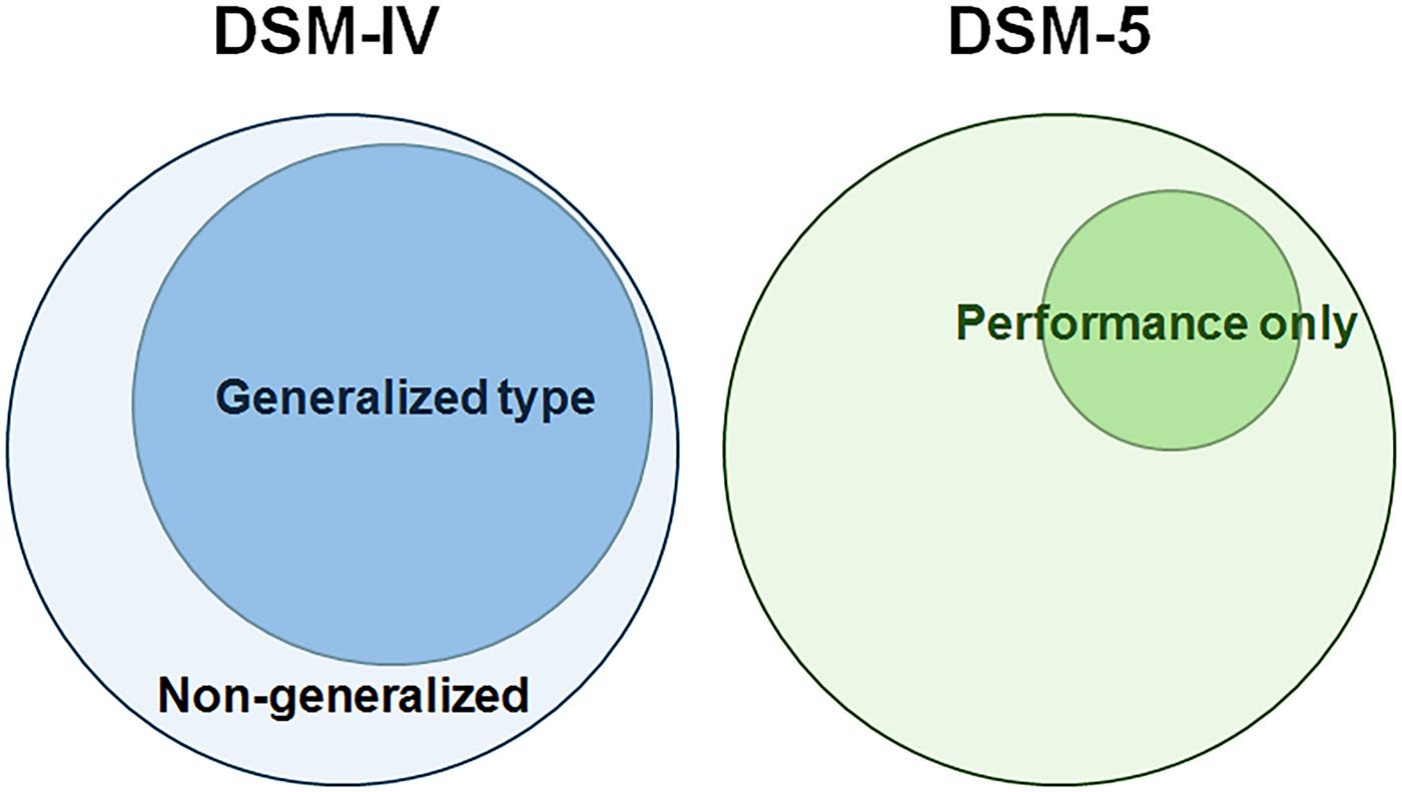
Changes in the specifiers of social anxiety disorder in the DSM-IV and DSM-5: From generalised and nongeneralized to performance-only.

In the previous DSM-IV criteria (APA 1994), the specifier for SAD was limited to the generalised type (Figure 1), which was defined as follows: “Generalised SAD is diagnosed when the individual’s fears include most social situations (e.g., initiating conversations, attending parties, speaking to authority figures).” (Table 1). On the basis of the limited supporting evidence for the generalised type and data substantiating that social anxiety symptomatology appears to fall along a continuum of severity, the DSM-5 Anxiety, Obsessive–Compulsive Spectrum, Posttraumatic, and Dissociative Disorders Work Group suggested the usefulness of a new specifier (Bögels et al. 2010; Heimberg et al. 2014). In the DSM-5 (APA 2013), the generalised type of SAD was removed, and the new “performance only” specifier for SAD was introduced to characterise individuals whose anxiety is limited to speaking or performing in public, emphasising a narrower range of the condition in a subgroup of persons (Figure 1). This was defined as follows: “Performance only SAD is diagnosed when the fear is restricted to speaking or performing in public.” “Individuals with performance-only SAD do not fear or avoid non-performance social situations” (Table 1). In the DSM-5, “SAD without the performance-only specifier” fully encompasses the generalised type described in the DSM-IV, meaning that individuals meeting the DSM-IV’s generalised type criteria are entirely included within this broader group.

**Table 1.** DSM-IV and DSM-5 specifications for social anxiety disorder.

DSM edition	Specifier	Definition
DSM-IV (APA 1994)	Generalised type	Generalised SAD is diagnosed when the individual’s fears include most social situations (e.g., initiating conversations, attending parties, speaking to authority figures).
DSM-5 (APA 2013)	Performance-only	Performance-only SAD is diagnosed when the fear is restricted to speaking or performing in public. Individuals with performance-only SAD do not fear or avoid non-performance social situations.

DSM, Diagnostic and Statistical Manual of Mental Disorders; APA, American Psychiatric Association; SAD, social anxiety disorder.

In terms of psychiatric nosology, it is also important to consider whether the performance-only specifier may, in some respects, overlap conceptually with specific phobia rather than representing a distinct subtype of SAD. Historically, when social phobia was first introduced as a diagnostic category in DSM-III (APA 1980), it primarily referred to situationally specific, performance-related fears, such as public speaking, eating or drinking in front of others, or urinating in public restrooms. In contrast, more pervasive and generalised forms of social anxiety were typically classified as avoidant personality disorder at that time. In subsequent DSM revisions, the diagnostic boundaries of social phobia were broadened, and comorbidity with avoidant personality disorder was permitted, resulting in a more heterogeneous clinical construct. Within this historical framework, the DSM-5 performance-only specifier reflects both diagnostic refinement and ongoing nosological ambiguity. This historical context provides an important basis for understanding the conceptual position of the performance-only subtype in relation to other anxiety disorders, including specific phobia.

The removal of the generalised type in the DSM-5 occurred because research revealed that the distinction between generalised and nongeneralized types lacked sufficient clinical utility (Ruscio et al. 2008; Peyre et al. 2016). The generalised type in the DSM-IV encompasses a broad range of social situations, making it difficult to apply consistently in diagnosis (Peyre et al. 2016). As a result, psychiatrists found it more practical to focus on the specific situations that trigger social anxiety, leading to a shift away from generalised categorisation. The introduction of the “performance only” specifier in the DSM-5 was driven by findings indicating that a subset of individuals experience anxiety exclusively in performance-related situations, such as public speaking or performing (Blöte et al. 2009; Aune et al. 2023; Rose and Tadi 2024). This subset often presents distinct characteristics, including specific triggers and unique treatment needs (Blöte et al. 2009; Heimberg et al. 2014; Peyre et al. 2016). By clearly defining the “performance only” specifier, the DSM-5 aimed to improve diagnostic precision and facilitate more targeted interventions for individuals whose anxiety is restricted to performance situations.

The objective of this narrative review is to summarise and compare current evidence on epidemiology, genetics, biological background, neuroimaging findings, clinical features, and treatment between SAD as a whole (referred to as overall SAD in this review), including both specifiers—generalised SAD and performance only SAD—according to DSM-IV and DSM-5, and performance-only SAD. By integrating findings across multiple domains, this review aims to clarify similarities and differences between overall and performance-only SAD and to highlight unresolved issues that warrant further investigation.

## Methods

### Literature search and study selection

This narrative review synthesised published evidence on overall SAD and performance-only SAD across multiple research domains. A literature search was conducted using PubMed and Web of Science to identify relevant articles published up to August 2025. Search terms included combinations of keywords related to social anxiety and performance-related anxiety, such as “social anxiety disorder,” “social phobia,” “performance-only,” “public speaking anxiety,” “epidemiology,” “genetics,” “neuroimaging,” “clinical features,” and “treatment.” In addition, the reference lists of key articles were manually screened to identify further relevant publications. Studies were selected based on their relevance to the scope of this review, with a focus on human studies published in peer-reviewed journals. A broad range of study designs was considered, including epidemiological surveys, family and twin studies, neuroimaging investigations, and clinical trials. Articles addressing either overall SAD, performance-only SAD, or closely related performance-based social fears were included. The identified literature was organised and narratively synthesised across key thematic domains, including epidemiology, genetics, neuroimaging, clinical features, and treatment. Emphasis was placed on comparing findings related to overall SAD and performance-only SAD, as well as on identifying areas of convergence, divergence, and gaps in the existing literature.

### Epidemiology of overall SAD and performance-only SAD

The lifetime prevalence of overall SAD is estimated to be approximately 5% to 13% in the general population (Burstein et al. 2011; Grant et al. 2005; Kessler et al. 2005; Ruscio et al. 2008; M. B. Stein 2006; Westenberg 1998), making it one of the most common anxiety disorders. The lifetime prevalence is comparable to that of specific phobias, which is estimated to be approximately 13% (Kessler et al. 2005). In contrast, the lifetime prevalence of performance-only SAD appears to be substantially lower when defined strictly according to the DSM-5 performance-only specifier, with studies reporting lifetime prevalence rates of approximately 0.3% to 0.7% in the general population and accounting for less than 25% of overall SAD cases (Blöte et al. 2009; Burstein et al. 2011; Crome et al. 2015; Peyre et al. 2016; Fuentes-Rodriguez et al. 2018). However, it is important to note that public speaking fear is highly prevalent in both the general population and among individuals with overall SAD, and prevalence estimates for performance-related anxiety vary considerably depending on how narrowly the subtype is defined. Epidemiological studies using broader definitions have reported higher proportions of individuals endorsing isolated public speaking fear, with estimates ranging from approximately 3% to 5% in community samples (M. B. Stein et al. 2000; M. B. Stein et al. 1996), and even higher proportions in mild forms of SAD identified through cluster-analytic approaches (Kessler et al. 1998; Furmark et al. 2000). Accordingly, from an epidemiological perspective, the key distinction lies in whether social anxiety is confined exclusively to performance situations or extends to a broader range of social contexts. While isolated public speaking fear may define a narrowly circumscribed performance-only presentation in some individuals, it more commonly occurs as part of a wider social fear profile within overall SAD. Taken together, these findings indicate that, when defined according to the DSM-5 performance-only specifier, performance-only SAD accounts for a relatively small proportion of overall SAD cases (Figure 1).

Overall SAD often begins in childhood or adolescence, with most cases developing in early adolescence (Rosellini et al. 2013). Approximately 50% of individuals experience onset by age 13, and approximately 90% experience onset by age 23 (Hofmann et al. 2004; Kessler et al. 2005; D. J. Stein et al. 2017; M. B. Stein 2006; Wong and Rapee 2015). Early onset, particularly before adolescence, is often associated with traits such as social reticence or behavioural inhibition. When SAD emerges at this early stage, these characteristics may contribute to its persistence or exacerbation as social and peer interactions become increasingly important during adolescence.

Although some studies have suggested that the performance-only subtype of SAD may emerge slightly later than overall SAD (Hofmann et al. 2004; Peyre et al. 2016; Fuentes-Rodriguez et al. 2018), both overall SAD and performance-only SAD typically show onset during adolescence, and age of onset alone does not appear to provide a clear distinction between performance-only SAD and overall SAD. The average age of onset for performance-only SAD has been reported to fall within adolescence or early adulthood (Bögels et al. 2010); however, this pattern should be interpreted as a tendency rather than a defining characteristic. Therefore, performance-only SAD may be less consistently associated with early developmental traits such as social reticence and behavioural inhibition, but potential differences in developmental trajectories across SAD subtypes remain to be clarified in future longitudinal studies.

There are notable gender differences in the prevalence of overall SAD. Compared with males, females are more likely to be affected by overall SAD (Blöte et al. 2009), with females experiencing SAD at approximately 1.5 times the rate of males (Crome et al. 2015; Peyre et al. 2016). Compared with females with overall SAD, males with overall SAD may experience more severe social and occupational impairments (Crome et al. 2015). Additionally, females with SAD often report a broader range of social fears, whereas males more commonly present with performance-related anxieties. With respect to performance-only SAD, sex differences are less pronounced than they are for overall SAD (Peyre et al. 2016). However, some studies suggest that the male-to-female ratio is higher among individuals with performance-only SAD than among those with overall SAD, particularly with respect to fears related to public speaking or performing (Peyre et al. 2016). Despite this, females still constitute the majority of individuals with performance-only SAD. Females with performance-only SAD may experience anxiety in a broader range of situations beyond public speaking, such as social events where they feel evaluated (Dell’Osso et al. 2015).

Furthermore, compared with individuals with overall SAD, individuals with performance-only SAD are more likely to be married, have attained a higher level of education, and report higher individual and family incomes (Peyre et al. 2016).

### Genetic differences between overall SAD and performance-only SAD

Family studies suggest that overall SAD shares common genetic vulnerabilities with other anxiety disorders, such as panic disorder and generalised anxiety disorder, indicating that genetic factors overlap across anxiety disorders (Li et al. 2011; Khanal et al. 2025). Additionally, family studies have shown that the prevalence of overall SAD is approximately 25% among individuals with a first-degree relative who has SAD, whereas it is 5% among those without such a family history (Bögels et al. 2010; M. B. Stein et al. 1998), highlighting the substantial influence of genetic factors on overall SAD.

In contrast, studies have reported no significant difference in the prevalence of performance-only SAD between individuals with and without a family history of overall SAD (M. B. Stein et al. 1998). This weaker familial aggregation suggests that genetic factors may be less strongly involved in the development of performance-only SAD than in overall SAD (Blöte et al. 2009). Accordingly, compared with overall SAD, environmental factors may play a relatively greater role in shaping the risk for performance-only SAD. Whereas overall SAD appears to arise from an interplay of genetic and environmental influences, including familial vulnerability and broader social experiences, performance-only SAD is more situationally specific and may be more strongly influenced by environmental exposures related to performance contexts, such as adverse experiences during public speaking, acting, or singing (Brook and Schmidt 2008; Blöte et al. 2009).

With respect to heritability, twin studies indicate that the heritability estimates for overall SAD range from approximately 30% to 60% (Middeldorp et al. 2005; Mosing et al. 2009; Scaini et al. 2014). By contrast, empirical data directly estimating the heritability of performance-only SAD are scarce. However, on the basis of family studies demonstrating weaker familial aggregation of performance-only SAD, it is reasonable to hypothesise that the heritability of performance-only SAD may be relatively lower than that of overall SAD. Importantly, this inference should be interpreted cautiously, as family studies do not provide direct estimates of heritability. Rather than indicating an absence of genetic influence, these findings may reflect a distinct aetiological balance, in which genetic factors play a relatively smaller role and environmental or situational triggers exert a proportionally greater influence in performance-only SAD compared with overall SAD.

In addition to differences in the overall strength of genetic influence, it is also possible that the genetic architecture underlying performance-only SAD is distinct in nature from that of overall SAD. Performance-only SAD has often been conceptualised as being more strongly shaped by conditioning processes, such as aversive performance-related experiences and associated cognitive or interpretive biases. However, fear conditionability itself may be influenced by genetic factors, and the genetic mechanisms involved in conditionability may differ from those underlying more broadly heritable anxiety-related traits. In light of these considerations, overall SAD has been shown to share genetic vulnerability with personality traits such as neuroticism and introversion, which are moderately heritable and associated with generalised negative affectivity. By contrast, performance-only SAD may be less strongly linked to these traits and instead involve genetic influences related to learning processes, threat sensitivity, or context-specific fear acquisition. Although these considerations remain speculative, taken together they suggest that performance-only SAD may be characterised by a distinct genetic profile rather than simply a lower degree of heritability. Future genetic research would benefit from systematically examining genetic correlations between SAD subtypes and other anxiety disorders, particularly specific phobia, as well as relevant personality traits. To date, most genetic studies of SAD have analysed the disorder as a single, undifferentiated phenotype, which has limited the ability to identify subtype-specific genetic mechanisms and to clarify whether distinct genetic pathways underlie overall SAD and performance-only SAD.

### Neuroimaging in overall SAD and performance-only SAD

Neuroimaging studies of individuals with overall SAD have revealed both structural and functional brain alterations associated with this disorder. Structurally, individuals with overall SAD exhibit differences in grey and white matter, influenced by age, comorbidities, and illness onset. In subcortical regions, compared with healthy controls, adults with SAD have been shown to have smaller bilateral putamen volumes and larger bilateral pallidum volumes. However, these volumetric differences are not observed in adolescents with SAD (Groenewold et al. 2023). Cortical alterations in individuals with SAD include reduced cortical thickness in areas such as the anterior cingulate cortex and insula, as well as increased cortical surface area, particularly in the frontal and temporal cortices (Bas-Hoogendam et al. 2018; Zhang et al. 2020). These findings suggest that cortical thickness and surface area may reflect distinct neurobiological mechanisms that jointly contribute to the emotional and cognitive symptoms of SAD. With respect to white matter integrity, individuals with overall SAD have shown reductions in major tracts, including the uncinate fasciculus (Phan et al. 2009; Baur et al. 2011), superior longitudinal fasciculus (Baur et al. 2011; Tükel et al. 2017), and inferior longitudinal fasciculus (Tükel et al. 2017). Increased radial diffusivity and mean diffusivity within these tracts indicate poorer myelination and neural disorganisation (Baur et al. 2011; Tükel et al. 2017). In particular, disruptions in the uncinate fasciculus may contribute to heightened fear responses and emotional dysregulation.

Resting-state functional imaging (rs-fMRI) studies have revealed altered intrinsic functional connectivity in individuals with overall SAD, particularly within the salience network (SN). Abnormal connectivity patterns have been reported between the amygdala and frontal (e.g., dorsomedial prefrontal cortex [dmPFC]), parietal (e.g., precuneus, posterior cingulate cortex), and temporal regions. These findings suggest variability in emotion processing and regulatory functions (Liu et al. 2015; Yoon et al. 2016; Jung et al. 2018). Disruptions in the temporal–amygdala network, particularly involving the fusiform gyrus, are less consistently observed and may reflect impairments in the processing of socially salient stimuli (Frick et al. 2013). However, findings across studies vary, especially concerning the direction and strength of amygdala connectivity. These inconsistencies may partly result from the exclusive use of resting-state paradigms. In contrast, task-based fMRI—typically involving social or anticipatory stress—may reveal connectivity patterns that are not observable at rest, accounting for some of the discrepancies among studies.

Although direct comparisons of task-based neuroimaging across SAD subtypes are currently lacking, several fMRI studies using social evaluative tasks have demonstrated increased limbic reactivity and altered fronto–limbic regulation in individuals with SAD. For example, patients with SAD show sustained and heightened amygdala activation during anticipation of public speaking compared with healthy controls (Davies et al. 2017), along with reduced regulatory coupling between prefrontal cortical regions and the amygdala during speech anticipation (Cremers et al. 2015). Furthermore, fMRI paradigms involving social conditioning have demonstrated exaggerated amygdala responses to socially salient cues, suggesting a neurobiological substrate underlying heightened threat sensitivity in social anxiety (Bas-Hoogendam et al. 2020).

In contrast to overall SAD, no structural or fMRI studies have specifically examined the neural correlates of performance-only SAD. Therefore, its neurobiological basis has not yet been directly characterised. On the basis of its situational specificity and generally milder clinical features, it is hypothesised that individuals with performance-only SAD may exhibit context-dependent amygdala hyperactivity during performance-related tasks, while showing largely typical neural patterns outside performance contexts. However, this remains speculative and highlights the need for targeted neuroimaging studies.

### Clinical features in individuals with overall SAD and individuals with performance-only SAD

Overall SAD and performance-only SAD share key clinical features (Boyers et al. 2017) but differ in the range and specificity of anxiety-provoking situations (Peyre et al. 2016; Fuentes-Rodriguez et al. 2018). Overall SAD involves a pervasive fear of social interactions, including conversations, meeting unfamiliar people, or being observed while performing daily tasks, leading to more severe impairments in social and occupational functioning (Boyers et al. 2017). Overall SAD exists on a continuum, ranging from absent or low social anxiety symptoms in healthy individuals to the most severe cognitive and behavioural symptoms in those with SAD. The number of feared social situations is positively associated with the severity of overall SAD (Peyre et al. 2016).

In contrast, performance-only SAD is limited to specific situations, such as public speaking, where the fear is focused on being evaluated in a particular setting. Individuals with performance-only SAD represent an exception to the continuum model (Peyre et al. 2016). Notably, performance-only SAD differs meaningfully from mild SAD, as those with the performance-only specifier experience highly situation-specific fear rather than generalised social anxiety across multiple contexts. Those with performance-only specifiers tend to have better mental health than those with generalised SAD, including a lower lifetime prevalence of mood and anxiety disorders, as well as certain personality disorders (i.e., obsessive–compulsive, paranoid, schizoid, schizotypal and borderline personality disorders), and lower rates of psychiatric disorders in the past 12 months (i.e., major depressive episode, mania or hypomania, and all anxiety disorders except for posttraumatic stress disorder). They also report higher quality of life and better social functioning compared to individuals with overall SAD (Kessler et al. 1998; Peyre et al. 2016; Fuentes-Rodriguez et al. 2018). Individuals with performance-only SAD experience a less persistent and less impairing subtype of overall SAD at the group level (Kessler et al. 1998; Fuentes-Rodriguez et al. 2018). However, it is important to recognise that performance anxiety can nonetheless be profoundly disabling for some individuals, particularly those whose occupations require frequent public performance, such as actors, musicians, teachers, or athletes. Accordingly, performance-only SAD can still lead to substantial distress in demanding or highly evaluative situations, such as delivering a speech in front of an audience.

### Treatments for overall SAD and performance-only SAD

Treatment for overall SAD is guided primarily by major clinical guidelines, including those from the UK National Institute for Health and Care Excellence (NICE, www.nice.org.uk/guidance/cg159), the German S3 (Bandelow et al. 2022), and the Japanese Society of Neuropsychopharmacology (JSNP) guidelines (Asakura et al. 2023). These guidelines emphasise cognitive behavioural therapy (CBT) as the first-line treatment, particularly the Clark & Wells (Clark and Wells 1995) or Heimberg model (Heimberg 2002). When face-to-face therapy is unavailable, NICE and JSNP recommend CBT-based guided self-help, whereas the S3 guidelines also mention psychodynamic psychotherapy as an alternative if CBT proves ineffective.

With respect to pharmacological treatments, selective serotonin reuptake inhibitors (SSRIs), such as escitalopram, sertraline, and paroxetine, are the preferred first-line pharmacological options across all guidelines. Serotonin–norepinephrine reuptake inhibitors (SNRIs), such as venlafaxine, are also recommended as either first- or second-line treatments. The NICE guidelines suggest the use of monoamine oxidase inhibitors (MAOIs), such as phenelzine, for treatment-resistant patients, whereas the German S3 guidelines include pregabalin for patients who are unresponsive to first-line treatments. The Japanese guidelines do not strongly recommend alternative pharmacological options such as MAOIs or pregabalin. Treatment effects of pharmacotherapy are generally observed within 4–6 weeks, whereas CBT may require a longer period to achieve significant improvement. In severe cases, or when monotherapy is insufficient, a combination of CBT and medication is advised.

Benzodiazepines are generally discouraged because of their potential for dependence, although the German S3 guidelines allow for short-term emergency use. Tricyclic antidepressants (TCAs) lack proven efficacy in overall SAD and are not recommended by the NICE or S3 guidelines, whereas the Japanese guidelines take no clear stance. Antipsychotics, such as olanzapine and quetiapine, are also discouraged because of their ineffectiveness and potential adverse effects. Additionally, the NICE and the German S3 guidelines caution against using gabapentin or herbal remedies because of insufficient evidence. Beta-blockers, such as propranolol, are not recommended for overall SAD in any of the guidelines. The endocannabinoid system (ECS) has been proposed as a novel therapeutic target for overall SAD (Ahmed et al. 2022). Compounds such as cannabidiol (CBD) and fatty acid amide hydrolase (FAAH) inhibitors show promise in early studies but are not yet approved or included in clinical guidelines. Further trials are needed to confirm their efficacy and safety.

Because research specifically targeting performance-only SAD remains limited, treatment strategies are primarily informed by findings from studies on overall SAD. CBT remains the primary treatment, with a particular emphasis on exposure-based techniques aimed at gradually reducing sensitivity to performance-related anxiety. Adjunctive methods, such as relaxation training and virtual reality exposure, may further enhance treatment outcomes. Pharmacologically, beta-blockers are occasionally used to alleviate acute physical symptoms, such as trembling and a rapid heartbeat, during performance situations. However, their efficacy is generally limited to physical manifestations and does not address the core emotional symptoms of SAD. SSRIs and SNRIs are occasionally used when individuals with performance-only SAD present with more generalised or persistent anxiety symptoms, although robust evidence for their effectiveness in this specific subtype is lacking. Recent studies have also explored novel treatments, such as fasedienol (PH94B) nasal spray, for as-needed use in social and performance anxiety. Although the initial results are promising, further research is needed to establish its clinical utility and long-term safety (Liebowitz et al. 2016).

## Discussion

This narrative review highlights the distinctive features of performance-only SAD compared with overall SAD (Table 2). Compared with overall SAD, performance-only SAD is characterised by a narrower range of social fears, tends to emerge later in adolescence, and shows a relatively higher prevalence of male representation. Individuals with performance-only SAD also tend to have fewer psychiatric comorbidities, better social functioning, and higher socioeconomic status. However, important gaps remain in the current evidence base. Direct genetic evidence supporting lower heritability in performance-only SAD is lacking, and neuroimaging studies specifically targeting this subtype have not yet been conducted. Although it has been hypothesised that performance-only SAD may involve a lower degree of genetic influence and fewer alterations in brain structure and connectivity compared with overall SAD, these assumptions remain speculative and require empirical validation. With respect to treatment, performance-only SAD is commonly managed using exposure-based cognitive–behavioural therapy and, in some cases, beta-blockers. Nevertheless, robust evidence supporting the efficacy of these interventions specifically for performance-only SAD is limited, and treatment recommendations are largely extrapolated from studies of overall SAD. Taken together, the relative scarcity of research directly focused on performance-only SAD has resulted in substantial gaps in our understanding of its underlying mechanisms, genetic architecture, and treatment responsiveness. Future studies explicitly designed to investigate this subtype are needed to establish whether performance-only SAD represents a distinct clinical entity and to inform the development of more targeted and efficient therapeutic strategies.

**Table 2.** Comparative profile between overall SAD and performance-only SAD.

Feature	Overall SAD	Performance-Only SAD
Lifetime prevalence	5%–13%	0.3%–0.7% (<25% of overall SAD)
Age of onset	50% by age 13; 90% by age 23	Adolescence to early adulthood
Gender distribution	Females > Males (1.5:1)	Females still majority, but higher male ratio than overall SAD
Global functioning level	Lower (more impairment)	Higher (better education, income, marriage rates)
Genetic contribution	Moderate (heritability 30-60%)	Possibly lower (based on family studies)
Structural MRI differences	Smaller putamen, larger pallidum, reduced WM integrity (uncinate, longitudinal fasciculi)	No direct evidence: amygdala and mPFC involvement hypothesised (speculative)
Resting-state fMRI differences	Predominantly increased amygdala–prefrontal connectivity and reduced fronto-parietal and default mode network connectivity	No direct studies; possibly increased amygdala–frontal connectivity; minimal large-scale network disruptions (speculative)
Psychiatric comorbidities	More comorbidities	Fewer comorbidities
Treatment approaches	CBT, SSRIs, SNRI; combination therapy common	CBT (with emphasis on exposure), beta-blockers for situational symptom relief

SAD, social anxiety disorder; WM, white matter; mPFC, medial prefrontal cortex; CBT, cognitive–behavioural therapy; SSRI, selective serotonin reuptake inhibitor; SNRI, serotonin-norepinephrine reuptake inhibitor.

## Author contributors

KO supervised the entire project, wrote the manuscript and was critically involved in the design, and interpretation of the data. All authors were responsible for performing the literature review, and intellectually contributed to data interpretation. All authors contributed to and approved the final manuscript.

## Data Availability

The data will be made available upon request.
